# Substance use as a source of belonging, relief and recognition – a reflective lifeworld study in Sweden

**DOI:** 10.1080/17482631.2026.2762635

**Published:** 2026-11-12

**Authors:** Isabel Reyes Andersson, Sally Hultsjö, Felicia Åsberg, Karolina Freij

**Affiliations:** a Department of Psychiatry, Ryhov County Hospital, Jönköping, Sweden; b Department of Health, Medicine and Caring Sciences. Division of Nursing and Reproductive Health, Linköping University, Linköping, Sweden

**Keywords:** Mental health, phenomenology, qualitative research, social support, substance-related disorders

## Abstract

**Background:**

Substance Use Disorder (SUD) is a public health concern with substantial consequences for health, well-being, and social functioning. Despite research, gaps remain in understanding how SUD develops within an individual’s life context, particularly how early life experiences are understood in relation to later substance use.

**Purpose:**

To describe the meanings of early life experiences in relation to substance use among adults receiving care for substance use disorder.

**Method:**

Seven adults receiving outpatient addiction care in Sweden participated in in-depth interviews. Data were analysed using Reflective Lifeworld Research (RLR). Reporting followed the Consolidated Criteria for Reporting Qualitative Research (COREQ).

**Results:**

Substance use is described as becoming a familiar way of seeking relief, belonging, and recognition in the context of insecurity and insufficient support. Its everyday availability, experiences of being left without a meaningful response, and belonging with similar others are interrelated meanings. Relief and social connection coexist with vulnerability, suffering, and loss of control.

**Conclusions:**

Early life experiences in relation to substance use are characterised by the intertwining of familiarity, unmet needs for support, and the possibility of finding relief and belonging through substances. Care and prevention should attend to these meanings and support other sources of safety, recognition, and connection.

## Introduction

Substance Use Disorder (SUD) is a global public health concern with substantial consequences for health, quality of life, and social functioning (Krendl and Perry, 2023; Volkow & Blanco, 2023). SUD is defined as a problematic pattern of alcohol, tobacco, illicit drug, or prescription drug use leading to clinically significant distress or impairment (American Psychiatric Association, 2022). It is characterised by a heterogeneous and fluctuating course, often involving progressive loss of control whereby substance use may come to dominate everyday life (Morck et al., 2020; Volkow & Blanco, 2023). Globally, an estimated 209 million people live with alcohol dependence, and more than three million deaths annually are attributed to alcohol and drug use (World Health Organisation, 2024a, 2024b). In Sweden, approximately 300,000 individuals have alcohol dependence and 70,000 have problematic drug use (Public Health Agency of Sweden, 2024).

SUD is also associated with disrupted relationships, housing instability, reduced participation in education and employment, stigma, social exclusion, and barriers to adequate care (Krendl and Perry, 2023; Volkow & Blanco, 2023). Consequences extend to family members, who may experience emotional and physical exhaustion, self-doubt, and social vulnerability (Farooq & Farhad, 2025). SUD therefore represents an important challenge for mental health nursing, highlighting the need to understand substance use in relation to the contexts and experiences through which it develops and becomes part of everyday life.

### Background

The development of SUD is influenced by multiple genetic, psychological, neurobiological, and social factors (Hatoum et al., 2022; Nath et al., 2022; Volkow & Blanco, 2023). Genetic factors contribute substantially to vulnerability to substance use disorders, with heritability of alcohol dependence at approximately 50%, with higher estimates reported for opioid dependence (Goldman et al., 2005; Verhulst et al., 2015). Further, family environment and early life experiences play a central role in the development of SUD (Manhica et al., 2024; Skarstein et al., 2023). Parental SUD influences children’s attitudes, behaviours, and expectations surrounding substance use and is associated with an increased risk of later alcohol and drug problems (Marino et al., 2018). Children growing up with parental SUD often experience instability, emotional unpredictability, disrupted caregiving, and difficulties accessing support (Meulewaeter et al., 2022; Muir et al., 2023). Previous research has also shown that these children are at increased risk of mental health difficulties, social vulnerability, and substance use later in life (Monari et al., 2024).

Beyond these adverse outcomes, qualitative studies have described how parental SUD may shape the child’s everyday life through secrecy, uncertainty, shifting family roles, and a persistent need to adapt to changing circumstances (Jørgensen et al., 2025; Meulewaeter et al., 2022). Children often assume responsibilities beyond their age while simultaneously struggling to make sense of unpredictable parental behaviours and family dynamics (Muir et al., 2023). Previous research also suggests that when substance use is embedded within everyday family life, it may come to be perceived as a normal and expected part of the child’s social world (Hedges, 2012). Consequently, the influence of parental SUD extends beyond exposure to substances themselves and may shape how children understand relationships, safety, support, and what is perceived as normal in everyday life.

Adolescence represents a particularly sensitive developmental period in relation to substance use. Early initiation of substance use is associated with an increased risk of later SUD (Green et al., 2024). During adolescence, peer relationships become increasingly important for identity formation, belonging, and social development, while the influence of family may gradually diminish (Veenstra, 2025). Exposure to social environments where substance use is normalised, including peer contexts in which substance use is common, may further increase the likelihood of substance use through processes of social influence, social learning, and belonging (Keyzers et al., 2020). Research also indicates that adolescents often form friendships with peers who share similar experiences, behaviours, and social positions, while simultaneously influencing one another over time (Veenstra, 2025). Consequently, peer relationships may not only provide opportunities for substance use but also shape how substance use is understood and integrated into young people’s everyday lives.

The Swedish context combines a publicly organised healthcare and social support system with policies intended to limit substance-related harm. Alcohol policy includes a state retail monopoly, taxation, and restrictions on availability, against a Nordic history of heavy episodic drinking (World Health Organisation Regional Office for Europe, 2023). Swedish drug policy combines prevention, treatment, and social interventions with restrictions on supply and criminal penalties for illicit use and possession (Socialutskottet, 2026). This context provides a background for understanding encounters with support services, but the study does not evaluate policy effects. Participants aged 24–63 recall early life across different historical periods; current services and policies should therefore not be assumed to describe all of their childhood circumstances.

Nevertheless, pathways into SUD cannot be fully understood through exposure to risk factors alone. The transition from substance use to SUD may occur relatively rapidly (Marel et al., 2019). Although associations between early life experiences, parental SUD, peer environments, and later substance use are well established, the present study focuses on how these experiences are lived as interconnected and meaningful by adults receiving care for SUD. To deepen understanding of how pathways into SUD are experienced and understood, epidemiological and biological perspectives need to be complemented by individuals’ subjective experiences (Gresko et al., 2023). The contribution sought is a description of the relationships between these meanings, rather than the identification of additional risk factors or a causal account of SUD. Such knowledge may inform person-centred prevention and care. Therefore, the aim of this study was to describe the meanings of early life experiences in relation to substance use among adults receiving care for substance use disorder.

## Method

### Design

A descriptive phenomenological study was conducted using Reflective Lifeworld Research (RLR) (Dahlberg et al., 2008). The phenomenon in this study is early life experiences in relation to substance use, as retrospectively described by adults receiving care for SUD. The focus is on how substance use is experienced in early everyday relationships and situations, rather than on SUD as a diagnostic category or on childhood in general. Lifeworld theory provides the ontological and epistemological foundation: experience is understood through the inseparable relationship between a person and the world. Meanings are explored as they appear in lived experience, rather than treated as meanings deliberately assigned by individuals. RLR emphasises openness, bridling of pre-understanding, and attention to the phenomenon throughout interviewing and analysis. Analysis seeks a structure of essential meanings and constituents that express its variations (Dahlberg and Dahlberg, 2008, 2020).

### Sampling

A broad, phenomenon-relevant purposive invitation was used. In RLR, strategic sampling seeks relevant experiences and variation in how a phenomenon is lived, rather than statistical representativeness (Dahlberg et al., 2008, 2026b). Adults older than 18 years receiving addiction care were eligible. No additional restrictions were imposed concerning substance type or duration of addiction, so that people with different experiences could participate. Staff provided study information, and information was also available in waiting rooms, enabling eligible individuals to contact the researchers themselves. Thus, the invitation was purposive in its relevance to the phenomenon and broad in scope, while participation was self-selected.The seven individuals who agreed to participate constituted three women and four men, aged 24–63 years (mean 36.4). Six participants lived in apartments and one lived with their parents; four were single and three were cohabiting with a partner. Six had completed compulsory education, and two had completed vocational training. Two participants were studying, one was employed, one participated in work training, one was seeking employment, and two reported no current employment or studies. Substance use histories predominantly involved opioids, often in combination with other substances. Alcohol, amphetamines, and benzodiazepines were also reported. One participant was currently abstinent, with a history of opioid and polysubstance use.

### Setting

Data were collected in a medium-sized region in southern Sweden with approximately 370,000 inhabitants and three psychiatric clinics. The region has nine addiction outpatient clinics with approximately 650 enroled patients. Treatment is primarily provided in outpatient settings by multidisciplinary teams comprising physicians, registered nurses, assistant nurses, counsellors, occupational therapists, and psychologists. Healthcare services primarily address medical and psychiatric needs, prevention, and rehabilitation (Hälso- och sjukvårdslag, 2017), while social services provide social support, housing assistance, and other psychosocial interventions (Socialtjänstlag (SFS 2025:400), 2025).

Care is primarily provided on a voluntary basis (Hälso- och sjukvårdslag, 2017; Socialtjänstlag (SFS 2025:400), 2025). When voluntary care is insufficient due to serious risks to an individual’s health, life, or development, compulsory care may be provided under specific legislation concerning children and adolescents, adults with severe substance use problems, and individuals with severe mental illness (Lag med särskilda bestämmelser om vård av unga, 1990; Lag om psykiatrisk tvångsvård, 1991; Lag om vård av missbrukare i vissa fall, 1988).

### Data collection

Permission to conduct the study was obtained from the heads of the three psychiatric clinics. The last author (IRA) coordinated recruitment with the addiction outpatient clinics. Interested individuals contacted IRA directly, after which the time and location of the interview were arranged according to participants’ preferences to facilitate a safe and undisturbed setting. Six participants were interviewed by IRA at one of the three addiction outpatient clinics, and one was interviewed by telephone by FÅ.

Before each interview, a brief conversation was held to establish a comfortable interview setting, and written informed consent was obtained. Interviews began with the open-ended invitation, “Can you tell me how you experienced the beginning of your substance use?” (Dahlberg et al., 2008). The interview questions also included broader directing questions about everyday life and relationships: “What did a typical day look like for you during that period?”, “How did you experience your relationships at that time, and in what ways did they affect you?”, and “When did you experience your substance use as developing into SUD?”. These questions directed attention to areas of experience and are distinguished here from follow-up questions. Follow-up questions invited elaboration of a particular experience, for example, “Can you describe a situation?” and “What did you feel at that moment?”. This distinction clarifies the questions used without implying that all questions were non-directive. The purpose was to obtain concrete descriptions relevant to the phenomenon and deepen reflection on lived situations (Dahlberg et al., 2008, 2026b). Field notes were made after the interviews to support reflection and analysis.

After each interview, time was provided for joint reflection on emotions evoked during the conversation. Interviews lasted 44–75 minutes (mean 50 minutes), were digitally recorded, transcribed verbatim, and de-identified. Data were collected between October and December 2025.

### Analysis

The analysis followed RLR (Dahlberg et al., 2008), aiming to describe the essential meanings of early life experiences in relation to substance use and the constituents through which these meanings vary. Essential meanings refer here to the empirical structure of meaning of the phenomenon, rather than a philosophical analysis of the lifeworld itself.

The analysis followed a whole–parts–whole movement. First, all authors independently read the transcripts several times to gain familiarity with the data and an overall understanding. The material was then divided into meaning units comprising sentences or longer passages relevant to the study aim. Meaning units were compared for similarities and differences, grouped into clusters, and repeatedly discussed among the authors before being synthesised into an overall description of the essential meanings. In relation to this structure of essential meanings, constituents were developed to illuminate variations and nuances of the phenomenon and how these were expressed in the empirical material (Dahlberg et al., 2008, 2020, Dahlberg and Dahlberg, 2026a).

To strengthen the grounding in lived experience, each constituent was illustrated with quotations reflecting individual expressions of the phenomenon, as central to lifeworld phenomenological research (Dahlberg et al., 2008). Participants were assigned pseudonyms in the quotations. The constituents represent variations through which the structure of essential meanings becomes visible (Dahlberg and Dahlberg, 2008, Dahlberg and Dahlberg, 2026a).

Throughout the analysis, all authors followed the principle of *bridling*, maintaining an open and sensitive approach while critically reflecting on pre-understandings, including theoretical assumptions, personal beliefs, and prior experiences. This involved resisting premature understanding and maintaining a reflective and flexible analytical attitude, allowing the phenomenon to reveal its meaning on its own terms (Dahlberg and Dahlberg, 2008, 2020).

### Ethical considerations

Ethical approval for the study was obtained from the Swedish Ethical Review Authority (No. 2025-01589-01). The research was conducted in accordance with the ethical principles for medical research involving human participants outlined in the Declaration of Helsinki (World Medical Association, 2024). The processing of personal data complied with the General Data Protection Regulation (GDPR) and the principles of good research practice issued by the Swedish Research Council (2024). The study was conducted and reported following the Consolidated Criteria for Reporting Qualitative Research (COREQ) (Tong et al., 2007). Clinical trial registration was not applicable.

## Results

Early life experiences in relation to substance use are described through a structure of essential meanings and three interrelated constituents. The constituents express variations in how substance use becomes familiar, how unmet needs for support are experienced, and how belonging is found with similar others (Figure 1).

**Figure 1.**
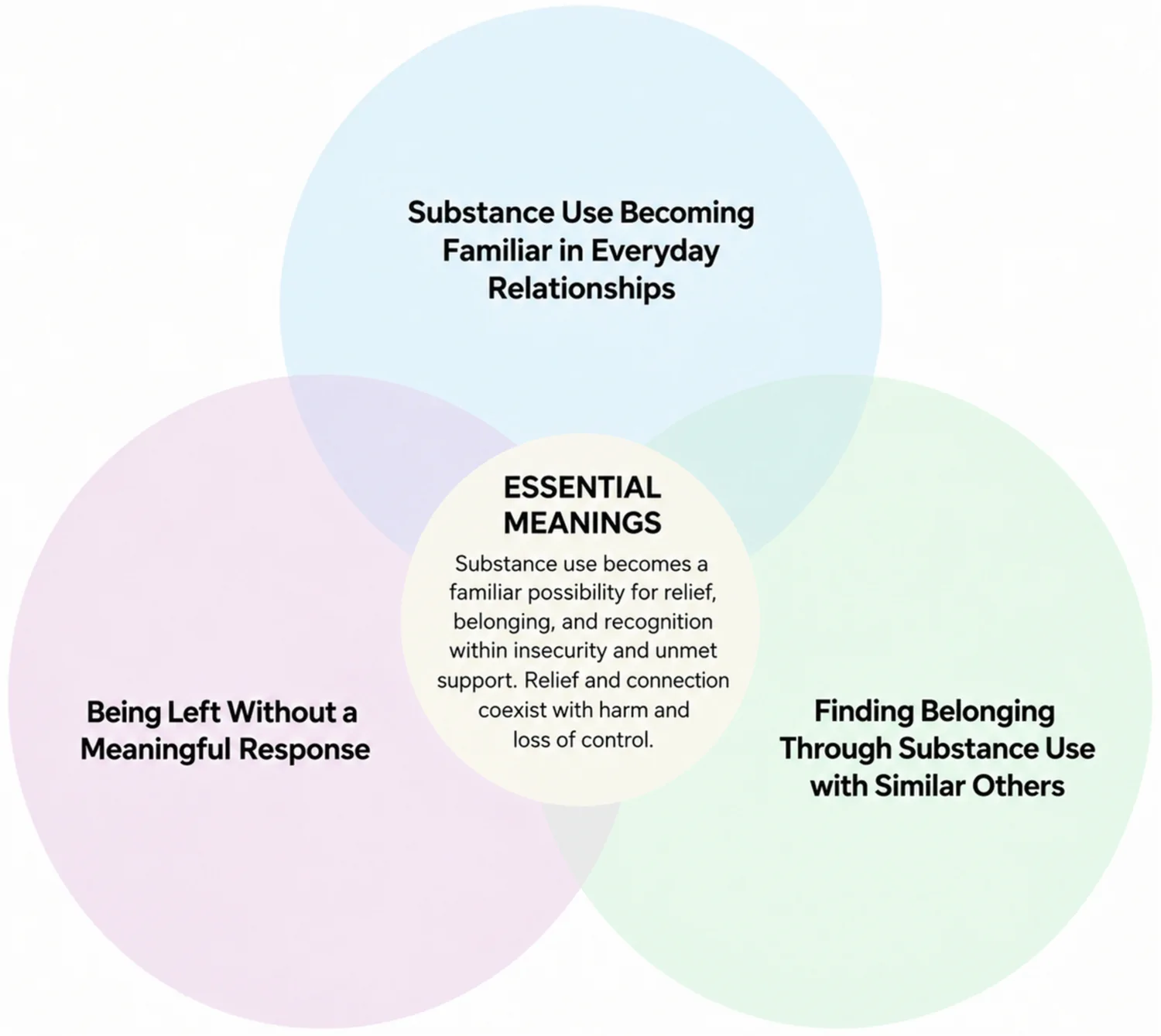
Essential meanings and interrelated constituents of early life experiences in relation to substance use.

### Essential meanings

Substance use becomes a familiar possibility for relief, belonging, and recognition within early experiences of insecurity and insufficient support. Its meaning is interwoven with everyday relationships: what offers a place among others can also expose one to harm. Familiarity with substances, being left without a meaningful response, and finding acceptance with similar others form an interconnected structure in which substance use becomes part of how everyday life is lived.

The availability of substances is inseparable from their experienced possibilities. Relief from distress and a sense of confidence or agency make substance use meaningful, while shared use offers connection and recognition. These possibilities coexist with suffering, vulnerability, and loss of control; relief does not remove harm, and belonging does not remove insecurity.

Early life experiences in relation to substance use are thus characterised by a tension between finding something needed and becoming exposed to further suffering. Substance use is described as gradually becoming embedded in familiar relationships and ways of coping, rather than as an isolated choice. Its significance lies in this intertwining of familiarity, unmet support, and the possibility of relief and belonging.

### Substance use becoming familiar in everyday relationships

Substance use in early life is described as familiar and available within everyday relationships. Encounters with alcohol, drugs, or medications occur through adults in the home, visiting adults, peers, and older adolescents. Curiosity and excitement coexist with being offered substances or finding them readily accessible. Familiarity does not require substance use within the immediate family; it can also develop through relationships outside the home. Experiences of parental use make consumption appear ordinary and expected, even when its extent would be regarded differently elsewhere. What becomes normal is therefore described through the relationships in which substance use is encountered.

*“… then what is abnormal becomes normal … so I have a mother who drinks, and drinks a lot, so you think it’s normal to drink that much. And then there is the hereditary factor, my mother was an alcoholic, my grandmother and grandfather were alcoholics.”* (Kim)

Becoming familiar with substance use involves being drawn into it through everyday relationships. Access to substances, offers from adults, and insufficient adult responsibility make one’s own use appear continuous with what is already known. Substances also offer experienced possibilities for courage, energy, confidence, and relief from anxiety. Familiarity and relief are thereby intertwined: substances are available not only as objects in the surroundings but as a way of making everyday situations more manageable.

*“I got to try benzos … because I remember that period when I got hold of them and started trying them, and it was like all the problems disappeared … and they took away the anxiety, they do, that’s what benzos do … But it was exactly that, that they took away the anxiety, that made me take them so much.”* (Charlie)

The familiarity of substance use coexists with insecurity. Conflict, parental separations, new adult relationships, emotional instability, and sometimes violence make home unpredictable. Substance use is thus described within relationships that can offer familiarity while failing to provide dependable care.

Insufficient attention to everyday needs, disordered home conditions, and a lack of response to emerging substance use deepen the experience of being without guidance. When substance-free adult relationships are absent, other ways of managing distress or organising daily life are less available within immediate experience.

### Being left without a meaningful response

Being left without a meaningful response is described as having difficulties that are visible without receiving dependable support. Parental SUD may be known to adults and authorities while everyday needs remain unattended until a crisis occurs. At school, concentration difficulties, outbursts, absence, unstable housing, and substance use can likewise be noticed without an adequate response. Visibility is then experienced through exclusion or failure rather than through understanding and help.

*“Well, yeah, there were difficulties concentrating, and back then you didn’t really get the help I would have needed at school, so there were a lot of outbursts and things like that … no one knew what to do, there weren’t any ADHD diagnoses back then, at least not around me. I definitely think it would have helped me.”* (Charlie)

The absence of a meaningful response gives visibility an ambivalent meaning. Encounters with teachers, social services, and other professionals can make difficulties known without bringing continuity, responsibility, or change. Being noticed does not in itself provide the experience of being understood or cared for.

In alternative residential placements, support is described as intertwined with fear, uncertainty, and lack of control. Abrupt moves, limited information about their purpose or duration, and uncertainty about contact with parents make intervention unpredictable. Degrading treatment and unclear reunification further intensify insecurity and a desire to escape.

*“… from when I was five, sometimes social services would just come and put you in a children’s home, you know … Yeah, I was scared then, you didn’t know why, or when is Mum coming to get you? You could end up staying there for six months … and then you weren’t allowed to see or talk to your mum or … and then one day Mum would just show up, like, ‘yeah, you’re coming home now’.”* (Alex)

The possibility of help remains present alongside the experience that it cannot be reached, even through repeated contact with authorities. Difficulties involving substance use, hygiene, and housing can remain without a sustained response. An apartment provided by social services can offer freedom while also being experienced as abandonment when schooling, daily routines, and substance use receive little attention. Retrospective descriptions express a wish for earlier, sustained intervention and a sense that other possibilities might then have opened.

### Finding belonging through substance use with similar others

Belonging through substance use is described as a movement towards people and settings that feel familiar and understandable. Shared experiences of instability, limited adult support, and substance use offer recognition. Encounters with peers and older adolescents make substances accessible, while shared use provides a place in a social world. Relationships with similar others feel self-evident, whereas different routines and expectations can seem distant or difficult to identify with.

*“No, no, I don’t think it was that much, it was more like … birds of a feather flock together … and you … automatically gravitate towards that kind of people … who are like-minded … so, yeah, we were a group who did it … at slightly different ages … I lived out in [city] then, grew up there … but then drugs came into it, of course.”* (Eliot)

Finding belonging with similar others also involves distance from unfamiliar ways of living. Stable daily routines can appear alien or even opposed to one’s own social world. In retrospective descriptions, this opposition is reconsidered, showing how what once feels familiar can later be understood differently.

*“… it was the first time I saw a normal person, or like, an ordinary person who, uh … got up in the morning and had breakfast and had lunch … no, I didn’t have any of that, you know … but I, I didn’t want to be part of what was normal either because I, I, uh … I had this really fucking distorted idea that it was the enemy …”* (Alex)

Belonging is described as possible in places and relationships otherwise associated with danger. When home offers conflict or unpredictability, acceptance among peers can feel safer and more dependable. Substance use is interwoven with this connection, enabling interaction while becoming taken for granted within everyday life. Participation offers identity, status, recognition, and a sense of significance that are difficult to find elsewhere. Safety and risk are therefore experienced together within the same relationships.

Belonging through substance use remains ambivalent. Anxiety, panic, physical discomfort, blackouts, withdrawal, and loss of control coexist with a wish to continue. Group loyalty includes caring for one another when risks arise, without involving adults. Continued participation can involve more intensive use, more potent substances, and access to selling or distributing substances. Connection and recognition are thus intertwined with vulnerability and exposure to further harm.

## Discussion

The essential meanings describe how familiarity with substances, unmet support, and belonging become intertwined in early lived experience. Rather than being merely a consequence of repeated exposure, normalisation emerges as substance use becomes embedded within everyday relationships, practices, and meanings. A central finding of the present study was that participants’ early exposure to substances gradually socialised them into a way of living in which substance use came to appear self-evident, contributing to a sense of having been born into a world where substance use was already present. Previous research similarly shows how family norms, values, and everyday interactions may contribute to substance use becoming perceived as ordinary and expected within family life (Hedges, 2012; Maina et al., 2021; Meulewaeter et al., 2022). Rather than understanding pathways into SUD primarily as the consequence of individual choices, the findings suggest that these pathways emerged within everyday relationships marked by substance use, insecurity, and limited opportunities for recognition (Hedges, 2012; Lindeman et al., 2022; Maina et al., 2021). From a phenomenological perspective, substance use may therefore become part of the horizon through which individuals come to understand themselves, others, and the world around them (Dahlberg et al., 2008). This raises the question not only of why some children develop substance use problems, but also of how alternative ways of living can become imaginable when substance use and instability form part of what is taken for granted.

The findings further indicate that participants’ everyday lives were characterised by insecurity, conflict, emotional absence, and a lack of stable adult guidance. These conditions were experienced not simply as isolated incidents but as recurring features of their lived worlds, shaping their sense of safety, belonging, and well-being. Previous research has demonstrated that parental SUD may compromise children’s access to consistent care and emotional support (Meulewaeter et al., 2022; Muir et al., 2023), thereby increasing vulnerability to mental health difficulties (Monari et al., 2024) and later substance use (Meulewaeter et al., 2022). The present study extends this knowledge by illuminating how such conditions become embedded in children’s everyday reality. Although developmental trajectories are shaped by multiple interacting factors (Kerr et al., 2024), early exposure to substance use may contribute to its normalisation and potentially to its reproduction across generations (Meulewaeter et al., 2022; Muir et al., 2023).

A related finding was the persistent experience of being visible yet unseen. Participants described circumstances in which school absenteeism, behavioural difficulties, parental substance use, unstable housing, and early substance use were known to adults and authorities, but where recognition rarely translated into sustained or meaningful support. Contact with schools, social services, and other welfare institutions seldom resulted in experiences of being understood or in changes to their everyday circumstances. Similar findings have been reported by Lindeman et al. (2022), who described how families affected by problematic substance use may experience themselves as invisible and insufficiently supported despite significant and long-standing difficulties. School absenteeism is associated with adverse educational and employment outcomes (Cattan et al., 2023; Hale & Viner, 2018). Early SUD is also associated with subsequent exclusion from education, employment, or training (Manhica et al., 2023). The relationship is also bidirectional, as high absenteeism may increase the risk of substance use while substance use may contribute to continued school absence (Gakh et al., 2020). These findings underline the importance of early identification and targeted interventions (Cattan et al., 2023; Manhica et al., 2023).

Schools and other social contexts may provide stability, emotional support, and relationships with trusted adults for children growing up with parental SUD (Jørgensen et al., 2025; Wangensteen et al., 2019). Such support may also be available through contexts such as sports associations, leisure activities, and digital platforms or groups (Ahlborg et al., 2024). However, children of parents with SUD may experience stigma related to their parents’ substance use, contributing to social isolation and creating additional barriers to seeking or receiving support. Coordinated efforts between schools, healthcare services, and social services are therefore important to identify, understand, and respond to the needs of these children (Allen et al., 2022; Jørgensen et al., 2025; Nkobi & Kingan, 2025). The present findings point beyond questions of service availability and raise questions about society’s capacity to respond when children’s suffering is already known. From a lifeworld-phenomenological perspective, relationships and everyday situations are central to how individuals experience health and well-being (Dahlberg et al., 2008). The experience of being recognised but not meaningfully supported may therefore shape children’s possibilities for developing trust, security, and alternative ways of understanding themselves and their circumstances.

Another central finding was that participants were drawn towards others with similar experiences of substance use, instability, exclusion, and limited adult support. Peer substance use predicts adolescents’ subsequent substance use, while peer relationships may shape substance use through processes of both selection and socialisation (Veenstra, 2025; Watts et al., 2024). However, the present findings suggest that the significance of these relationships extends beyond exposure to substances. Rather than representing simply a search for deviant peers, participants appeared to move towards people and environments that felt familiar, understandable, and consistent with their own lived experiences. Previous research similarly suggests that substance use may serve important social functions during adolescence by facilitating access to friendships and social contexts in which young people experience belonging and acceptance (Poulin et al., 2011). Substance use thus acquired meaning through the relationships and communities in which it occurred, becoming intertwined with efforts to establish social connection and a sense of place in the world, even when these contexts also entailed increased vulnerability and risk.

The findings further reveal the fundamentally ambivalent nature of substance use. Participants described substances as both relieving and harmful, empowering and destructive. Substance use was experienced as alleviating anxiety, emotional distress, and social insecurity while facilitating social participation, confidence, and a sense of agency. Initial physiological effects of substances are often perceived as pleasurable, whereas negative reactions may include physical discomfort such as nausea and dizziness (Kingston et al., 2017). At the same time, substance use may function as a means of coping with emotional distress and creating a sense of belonging (Rich, 2020), and has been associated with increased openness, self-confidence, and emotional regulation (Meulewaeter et al., 2022). These perceived effects may help explain why substance use can become meaningful despite its harmful consequences. Some participants also described substance use in relation to self-medication, including in the context of undiagnosed ADHD. ADHD is associated with earlier substance use initiation and more severe SUD trajectories, highlighting the importance of early and comprehensive support (Rohner et al., 2023).

These findings illustrate that experiences of substance use are simultaneously bodily, psychological, and relational. Individuals experience and interpret the effects of substances through their bodies, influencing whether substances are experienced as contributing to safety and well-being or to physiological discomfort and suffering (Dahlberg et al., 2008). From a lifeworld perspective, these apparently contradictory meanings should not be understood as mutually exclusive. Rather, they demonstrate how individuals may use the possibilities available to them to manage suffering and create moments of relief, belonging, and agency. Substance use may therefore be understood not solely as irrational or self-destructive behaviour, but also as an attempt to make difficult circumstances more bearable and meaningful. This interpretation resonates with Dahlberg et al. (2008), who conceptualise well-being and suffering as interconnected dimensions of lived experience. The findings consequently challenge simplified understandings of substance use as either beneficial or harmful and highlight how its meaning is shaped by the circumstances in which it is lived.

### Limitations

A lifeworld phenomenological approach was chosen to describe the meanings of early life experiences in relation to substance use. The accounts are retrospective and are understood from participants’ present situations in addiction care; they do not provide direct observation of childhood or prospective evidence of how SUD develops. Phenomenological in-depth interviews require participants to articulate their experiences and reflections (Dahlberg et al., 2026b). At the beginning of each interview, the interviewer assessed participants’ capacity to engage, and no participant showed significant impairment, difficulties expressing their experiences, or signs of being under the influence of substances.

Interviews involve ethical considerations concerning dignity, protection from harm, and participants’ right to have their experiences heard (Swedish Research Council, 2024). The interviewer remained attentive to the possibility that interviews could evoke emotionally sensitive memories (Dahlberg et al., 2008, 2026b), and participants were given sufficient time for reflection and emotional processing.

Although a variation in age, gender, social circumstances and substances used the small sample limits the experiences relevant to the phenomenon. Recruitment depended on individuals responding to information in addiction services; people who did not volunteer or were not in treatment are not represented. The broad invitation allowed different experiences to be included, but did not constitute deliberate selection across predetermined groups. In RLR, depth and variation in meanings are important considerations when assessing empirical material (Dahlberg et al., 2008, Dahlberg and Dahlberg, 2026a, 2026b). Additional interviews might have challenged or extended the proposed structure of essential meanings. The findings should therefore be read as a contextualised description grounded in this material.

One interview was conducted by telephone, while the remaining interviews were conducted face-to-face. Although face-to-face interviews allow non-verbal expressions to contribute to understanding lived experience (Dahlberg et al., 2026b), the telephone interview was comparable in length and depth and increased accessibility.

The findings are grounded in lived-experience accounts and are not intended to be statistically generalisable (Dahlberg et al., 2008). Descriptions of recruitment, setting, and the research process enable readers to consider relevance to other contexts. Sweden’s welfare services and alcohol and drug policies, as well as changes over the periods recalled, may influence both the experiences described and their transferability. The study cannot distinguish policy effects or establish that the meanings described apply to all people with SUD.

## Conclusion

Early life experiences in relation to substance use are described through the intertwining of familiarity, unmet needs for support, and belonging with similar others. Substance use becomes meaningful as an available way to obtain relief, recognition, and agency within insecure relationships and situations. These possibilities coexist with harm and loss of control. The central contribution is this tension: a source of suffering can also be experienced as a source of something needed. Prevention and care should attend to these interconnected meanings and support alternative ways of experiencing safety, relief, and connection. The findings concern retrospective lived experience and do not establish a causal pathway to SUD.

### Relevance to clinical practice

All professional groups working with people with SUD, including healthcare professionals, social workers, and others providing support, should explore what substance use means to the individual and the functions it serves in everyday life to support person-centred care that addresses needs for safety, belonging, recognition, relief, and agency. Treatment should recognise that reducing substance use may involve losing relationships and social contexts that provide belonging and identity, highlighting the importance of supporting alternative sources of connection, participation, and recognition for sustainable recovery. For children and young people exposed to parental substance use and insecurity, early relational support should be prioritised, with prevention extending beyond substance use initiation to strengthening stable relationships, safety, belonging, and opportunities for recognition. Understanding the individual’s everyday relationships and life situation should inform this work. Coordinated support across mental health care, addiction services, schools, and social services may therefore be important for identifying and addressing these relational and social needs at an early stage.

## Data Availability

The data are not publicly available due to privacy and ethical restrictions related to the sensitive and potentially identifiable nature of the qualitative interview data.

## AI and AI-assisted technologies in the writing process

In this work, AI-assisted support through Linköping University’s Microsoft Copilot application was used in several ways. The tool was used, among other purposes, to improve and condense sections of the manuscript and to translate Swedish text into English throughout the manuscript. AI was also used to create the figure. OpenAI ChatGPT was additionally used to support language editing.
